# Protein disulfide isomerase expression increases in resistance arteries during hypertension development. Effects on Nox1 NADPH oxidase signaling

**DOI:** 10.3389/fchem.2015.00024

**Published:** 2015-03-27

**Authors:** Aline C. D. Androwiki, Lívia de Lucca Camargo, Simone Sartoretto, Gisele K. Couto, Izabela M. R. Ribeiro, Sidney Veríssimo-Filho, Luciana V. Rossoni, Lucia R. Lopes

**Affiliations:** ^1^Department of Pharmacology, Institute of Biomedical Sciences, University of São PauloSão Paulo, Brazil; ^2^Department of Physiology and Biophysics, Institute of Biomedical Sciences, University of São PauloSão Paulo, Brazil

**Keywords:** protein disulfide isomerase, hypertension, NADPH oxidase, Nox1, reactive oxygen species, mesenteric resistance arteries, aorta

## Abstract

NADPH oxidases derived reactive oxygen species (ROS) play an important role in vascular function and remodeling in hypertension through redox signaling processes. Previous studies demonstrated that protein disulfide isomerase (PDI) regulates Nox1 expression and ROS generation in cultured vascular smooth muscle cells. However, the role of PDI in conductance and resistance arteries during hypertension development remains unknown. The aim of the present study was to investigate PDI expression and NADPH oxidase dependent ROS generation during hypertension development. Mesenteric resistance arteries (MRA) and thoracic aorta were isolated from 6, 8, and 12 week-old spontaneously hypertensive (SHR) and Wistar rats. ROS production (dihydroethidium fluorescence), PDI (WB, imunofluorescence), Nox1 and NOX4 (RT-PCR) expression were evaluated. Results show a progressive increase in ROS generation in MRA and aorta from 8 to 12 week-old SHR. This effect was associated with a concomitant increase in PDI and Nox1 expression only in MRA. Therefore, suggesting a positive correlation between PDI and Nox1 expression during the development of hypertension in MRA. In order to investigate if this effect was due to an increase in arterial blood pressure, pre hypertensive SHR were treated with losartan (20 mg/kg/day for 30 days), an AT1 receptor antagonist. Losartan decreased blood pressure and ROS generation in both vascular beds. However, only in SHR MRA losartan treatment lowered PDI and Nox1 expression to control levels. In MRA PDI inhibition (bacitracin, 0.5 mM) decreased Ang II redox signaling (p-ERK 1/2). Altogether, our results suggest that PDI plays a role in triggering oxidative stress and vascular dysfunction in resistance but not in conductance arteries, increasing Nox1 expression and activity. Therefore, PDI could be a new player in oxidative stress and functional alterations in resistance arteries during the establishment of hypertension.

## Introduction

Among the factors involved in the pathophysiology of hypertension evidence indicates an important role of reactive oxygen species (ROS) (Pechanova et al., 2006). Several studies associate hypertension with high concentrations of ROS in animal models (Heitzer et al., 1999; Kitamoto et al., 2000; Beswick et al., 2001) and in humans (Lacy et al., 1998; Lee et al., 2003). Indeed, the increase in ROS in spontaneously hypertensive rats (SHR) may contribute to left ventricular hypertrophy, coronary artery disease (Shah and Channon, 2004), kidney damage (Adler et al., 2004), endothelial dysfunction (Rodrigues et al., 2006) and remodeling of pulmonary arteries (Demarco et al., 2008).

An important source of ROS in the cardiovascular system is the NADPH oxidase family of enzymes. These enzymes generate superoxide, that can reduce the bioavailability of nitric oxide (NO) as well as serve as a precursor for other species such as H_2_O_2_ and ONOO^−^ (Rey et al., 2001). The NADPH oxidase comprises a family of enzymes known as Nox that catalyze the reduction of molecular oxygen generating superoxide. Although all Nox isoforms are transmembrane proteins capable of generating ROS, they differ in distribution and cellular localization, association with regulatory subunits, ROS generated and activation mechanisms. These differences are responsible for the different functions of Nox and confer specificity to the action of ROS as second messengers, making the generation of these species a controlled and compartmentalized event. Unlike phagocytic NADPH oxidase, vascular NADPH oxidase can be regulated by gene transcription of its subunits. Thus, NADPH oxidase activity can be regulated through assembly of the regulatory subunits to the membrane or by increased expression of the enzyme (Brandes and Schroder, 2008).

In the cardiovascular system Noxes can regulate vascular function proliferation, migration and hypertrophy of vascular smooth muscle cells (VSMC) as well as cell differentiation and angiogenesis (Cave et al., 2006; Garrido and Griendling, 2009). Some aspects differ between Nox1 and Nox4 isoforms. Nox 4 is located in the endoplasmic reticulum and nucleus (Ambasta et al., 2004) and generates H_2_O_2_ (Takac et al., 2011) constitutively and independent of cytosolic subunits (Ellmark et al., 2005). On the other hand, in VSMC Nox1 is located in caveolae (Hilenski et al., 2004), plasma membrane and endosomes (Miller et al., 2010) and generates O^−^_2_. The generation of ROS by Nox1 can be stimulated by Ang II. Initially, the binding of Ang II to the AT1 receptor activates protein kinase C which phosphorylates p47phox. This initial phase of ROS generation is rapid and occurs within seconds after Ang II stimulation. Maintenance of Nox1 activation requires transactivation of EGFR and activation of ERK1/2 which leads to an increase in Nox1 expression and vascular smooth muscle cell proliferation (Stanic et al., 2010). In fact, several studies have shown that expression of Nox1 is increased in injured arteries of Wistar rats (Szocs et al., 2002), of diabetic rats (Wendt et al., 2005) and in SHR (Matsuno et al., 2005; Tabet et al., 2008). Furthemore, mice with deficiency of Nox-1 show a decrease in blood pressure (Gavazzi et al., 2006).

Studies from our group identified PDI as a protein which associates with NADPH oxidase subunits regulating Ang II dependent ROS generation in VSMC. Moreover, this association was also demonstrated in leukocytes (Santos et al., 2009; Paes et al., 2011) and endothelial cells (Laurindo et al., 2008).

Interestingly, Nox1 is associated with an increased generation of ROS in Ang II infusion hypertension models (Dikalova et al., 2005). Taken together, these studies indicate a close association between the expression of PDI and the expression and activity of vascular NADPH oxidases. Therefore, understanding the role of PDI in the activation of vascular NADPH oxidases in diseases such as hypertension may provide a new approach for the development of new forms of therapeutic intervention in the treatment of this disease. Thus, the goal of this study was to determine PDI expression and NADPH oxidase dependent ROS generation and signaling in conductance and resistance arteries during hypertension development.

## Materials and methods

### Animals and tissue preparation

All procedures carried out were approved by the Ethics Committee for Animal Experimentation of the Institute of Biomedical Sciences (ICB) at University of São Paulo (São Paulo, Brazil) in accordance with the guidelines from the Brazilian College for Animal Experimentation (COBEA) under Federal Law 11794. Male SHR and Wistar rats were housed in climatically controlled environment (12 h light–dark cycle at 22°C) with access to rodent chow and water *ad libitum*.

### Measurement of blood pressure

Systolic blood pressure was determined in conscious rats using an indirect tail-cuff method (pneumatic transducer, PowerLab 4/S, AD Instruments Pty Ltd), a few days before experimentation. Rats were preheated at 40°C for 5 min, and three stable consecutive measurements of blood pressure were averaged.

### Anti hypertensive treatment

In order to investigate the effect of AT1 activation in PDI expression during the development of hypertension, pre hypertensive, 7 week-old SHR and Wistar rats were treated with the AT1 receptor antagonist, Losartan (20 mg/kg/day) by gavage for 30 days (Koprdova et al., 2009). A separate group of animals received vehicle (water) and was used as control. Untreated or treated age- and weight-matched SHR or Wistar rats were decapitated and thoracic aorta and mesenteric arteries were removed. Afterwards, arteries were washed with ice-cold PBS, and maintained at 4°C in Krebs-Henseleit (KHS) solution (112.0 mM NaCl, 4.7 mM KCl, 2.5 mM CaCl_2_, 1.1 mM KH_2_PO_4_, 1.2 mM MgSO_4_, 25.0 mM NaHCO_3_, and 11.1 mM glucose). The samples were carefully dissected and processed according to the techniques described below.

### Detection of reactive oxygen species (ROS) generation

ROS generation was measured by detection of fluorescent dihidroethidium (DHE) oxidation products. In the presence of superoxide and other reactive species, DHE is oxidized to 2-Hydroxyethidium and ethidium, which are trapped by intercalation with DNA resulting in bright red fluorescence (excitation: 480; emission: 580 nm). Thoracic aorta and mesenteric arteries of SHR and Wistar rats were embedded in Tissue Tek O.C.T. Compound (Sakura Finetek, Torrance, CA, USA). Unfixed frozen cross sections (5 μm) were incubated with DHE (5 μmol/L; Molecular Probes) diluted in phosphate buffer with DTPA (100 μmol/L); in a light-protected and humidified chamber at 37°C for 30 min. A group of artery slices were treated with PEG-SOD (150 U/mL) for 30 min before incubation with DHE. Fluorescence was detected with a 490–590 nm long-pass filter, under a microscope (Axiovert, Zeiss) with a 20× objective lens coupled to a digital camera. Fluorescent images were recorded with exposure intensity adjusted relatively to 6 weeks Wistar rats. Fluorescent images were analyzed by measuring the mean optical density of the fluorescence in a computer system (Image J®). This fluorescence was evaluated in at least three different locations in each image and normalized by area.

### Quantitative PCR analysis

Mesenteric arteries and aortas were removed, frozen in liquid nitrogen and homogenized. RNA was isolated by trizol chloroform method. Synthesis of cDNA was carried out with MMLV (Promega). Quantitative PCR reactions were performed, recorded, and analyzed using the Corbett Research system (Corbett Life Sciences, Sydney, Australia). The specificity of the SYBR® green assay was confirmed by melting-point analysis. Relative expressions of targeted genes were calculated from the cycle threshold (Ct) value using the ΔCt method for quantification. Gene expression of hypoxanthine phosphoribosyltransferase mRNA (HPRT) was used for normalization. All oligonucleotides and reagents utilized in this protocol were purchased from Invitrogen (Carlsbad, CA, USA). Primer sequences were as followed:

Nox1: sense – 5′-TTCCCTGGAACAAGAGATGG-3′, and antisense – 5′-GACGTCAGTGGCTCTGTCAA-3′;
Nox4: sense – 5′-CTGGAAGAACCCAAGTTCCA-3′, and antisense – 5′-CGGATGCATCGGTAAAGTCT-3′;
HPRT: sense – 5′-TTTTGCTGACCTGCTGGATTAC-3′, and antisense: – 5′-TACTTTTATGTCCCCCGTTGA-3′.

### Immunofluorescence assay

Mesenteric arteries and aorta segments were embedded in Tissue Tek O.C.T. Compound (Sakura Finetek, Torrance, CA, USA). Tissues sections (7–14 μm) were obtained in a cryostat (Leica Microsystems, Germany, model CM1850) and mounted in gelatized slides. After fixing in paraformaldehyde 4% (30 min), sections were incubated with a blocking buffer containing 0.3% Triton X-100, 2% normal goat serum and 1% BSA for 2 h. Sections were then incubated overnight with anti-PDI antibody (anti-mouse anti-PDI 1:100; Thermo scientific, USA) diluted in blocking buffer followed by incubation with a FITC-coupled secondary antibody (goat anti-mouse – FITC, 1:200) at room temperature for 2 h. Controls for immunostaining included the omission of the primary antibody and its substitution for normal goat serum, which completely eliminated staining. Immunoreactivity was visualized by fluorescence microscopy (Axioskop, Zeiss) using a x20 objective lens coupled to a digital camera. Fluorescent images were recorded with exposure intensity adjusted relative to Wistar 6 weeks.

### Western blotting

Frozen mesenteric arteries and aortas were homogenized in cold lysis buffer [50 mmol/L of Tris/HCl (pH 7.4), 5 mmol/L of EGTA, 2 mmol/L of EDTA, 0.1 mmol/L of PMSF, 1 mmol/L of pepstatin A, 1 mmol/L of leupeptin, and 1 mmol/L of aprotinin]. Homogenates were centrifuged at 13000 g for 30 min. Equal amounts of proteins (20 or 75 μg) were loaded on a 10% sodium dodecyl sulfate polyacrylamide gel (SDS-PAGE), transferred onto a nitrocellulose membrane and incubated overnight at 4°C with primary antibody. Proteins were detected by infrared laser scanner using an Odyssey CLX 100 (Li-Cor Biosciences, Nebraska, USA). Densitometry was determined using the Odyssey package and quantified through computer analysis, using the Image J program (Molecular Dynamics, Sunnyvale, CA, USA). Antibodies used were: anti-PDI from Thermo Scientific (Rockford, IL, USA), anti- ERK 1/2 and phospho ERK 1/2 from Cell Signaling (Danvers, MA, USA), anti eNOS BD Transduction (NJ, USA), anti-β-actin and anti-α actin from Sigma Aldricht (St Louis, MO, USA). Results were normalized to total non phosphorylated protein or with α- or β-actin in the same sample run on the same gel/membrane.

### Statistical analysis

The results were expressed as mean ± S.E.M. Statistical comparisons were performed using One Way or Two Way analysis of variance (ANOVA) with appropriate *post-hoc* analysis. A value of *P* < 0.05 was considered significant.

## Results

### Hypertension development is accompanied by oxidative stress and increased Nox-1/ PDI expression only in mesenteric resistance arteries

**Figure 1:**
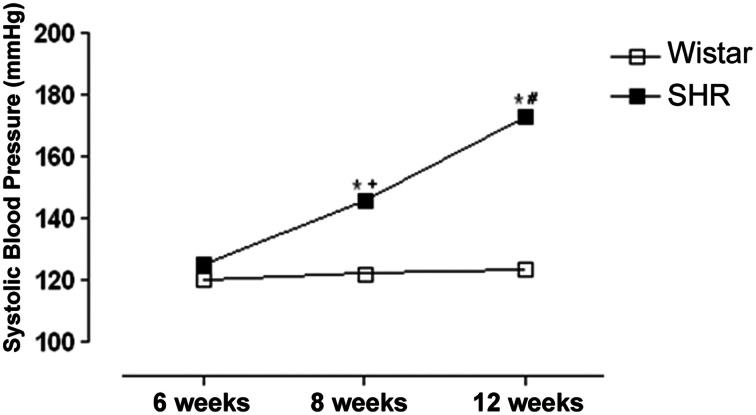
**Development of systolic blood pressure (SBP) increases in spontaneously hypertensive rats (SHR)**. SBP was measured by tail-cuff method in 6, 8 and 12 week-old SHR and Wistar rats. Results are expressed as mean ± SEM of *n* = 5 animals. ^*^p < 0.05 vs. Wistar; ^+^*p* < 0.05 vs. SHR 6 weeks and ^#^*p* < 0.05 vs. SHR 6 and 8 weeks.

Figure 1 shows that SHR were normotensive at 6 weeks of age; however, at 8 weeks of age SHR presented an increase in blood pressure as compared to Wistar rats, which was even higher at 12 weeks and this stage was considered a phase of established hypertension.

**Figure 2:**
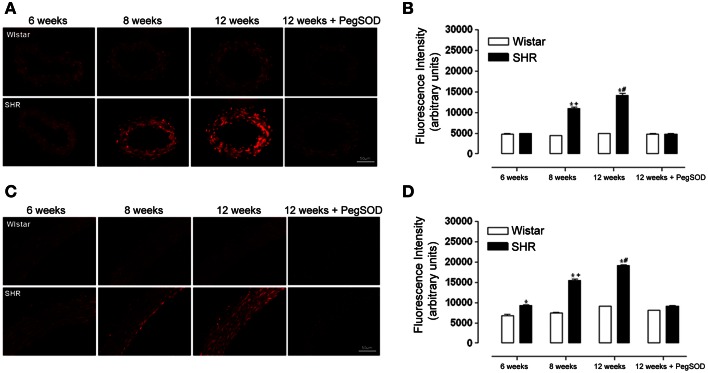
**ROS generation increases during hypertension development in mesenteric resistance arteries and aorta from hypertensive rats**. Analysis of dihydroethidium (DHE; 5 μmol/L) derived fluorescence **(A,C)** and quantification **(B,D)** in slices of mesenteric resistance arteries **(A,B)** and aorta **(C,D)** of 6, 8, and 12 week-old SHR and Wistar rats. A set of serial sections were treated with PEG SOD (150 U/mL) for 30 min before incubation with DHE and used as experiment control. Images are representative of three different experiments. Images were acquired using a 20× objective lens with exposure intensity adjusted relatively to 6 weeks Wistar. Results are expressed as mean ± SEM of *n* = 3 experiments. ^*^p < 0.05 vs. Wistar; ^+^*p* < 0.05 vs. 6 weeks SHR; and ^#^*p* < 0.05 vs. 6, 8, and 12 weeks SHR + PegSOD.

ROS generation increased in 8 and 12 week-old SHR in both mesenteric resistance arteries (Figures 2A,B) and aorta (Figures 2C,D). Interestingly, ROS generation in aorta of SHR was already increased at 6 weeks when the animals were still normotensive (Figures 2C,D).

**Figure 3:**
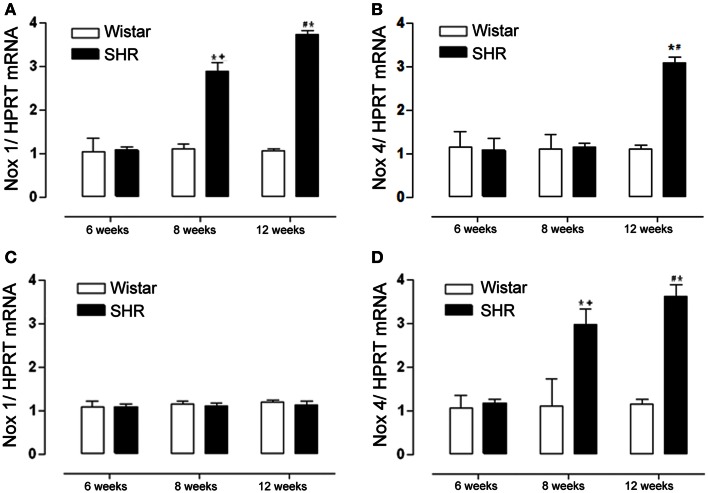
**Nox1 but not Nox4 expression increases only in mesenteric resistance arteries during hypertension development**. Nox1 and Nox4 mRNA levels were detected by RT-PCR. Nox1 mRNA expression was increased in mesenteric arteries **(A)** from 8 and 12 weeks SHR while in aorta **(C)** there was no difference among groups. Nox4 mRNA expression was increased in mesenteric arteries **(B)** from 12 weeks SHR, while in aorta **(D)** Nox4 was increased in 8 and 12 weeks SHR. Results were normalized to HPRT mRNA and are expressed as fold increase as compared to 6 weeks Wistar. Results are expressed as mean ± SEM of *n* = 5 experiments. ^*^p < 0.05 vs. Wistar ^+^*p* < 0.01 vs. SHR 6 weeks; ^#^*p* < 0.05 vs. SHR 6 and 8 weeks.

A progressive increase in Nox1 expression was observed in mesenteric resistance arteries from 8 to 12 weeks in SHR (Figure 3A). On the other hand, in aorta there was no difference in Nox1 expression between SHR and Wistar rats in any of the age-matched animals studied (Figure 3C). Nox 4 expression was increased only in 12 week-old mesenteric resistance arteries (Figure 3B), while in aorta there was a progressive increase from 8 to 12 weeks in SHR (Figure 3D).

**Figure 4:**
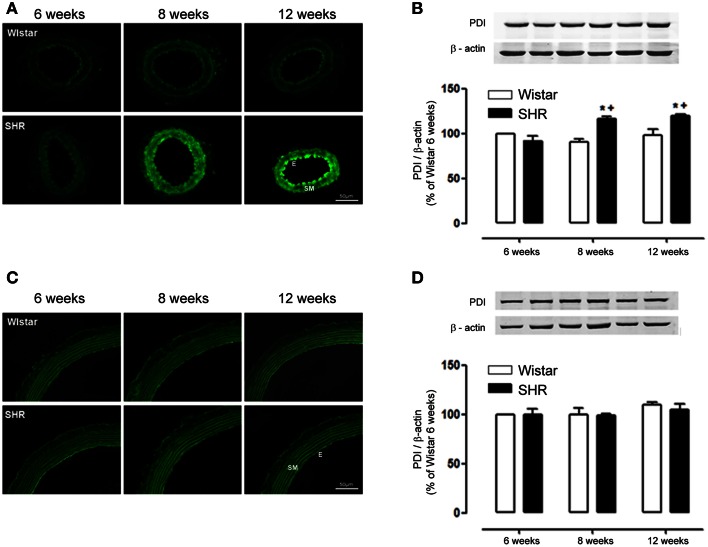
**PDI expression increases only in mesenteric resistance arteries during hypertension development**. In mesenteric arteries, PDI expression detected by immunofluorescence (**A**, green) and western blot **(B)** was increased in 8 and 12 week-old SHR. In aorta, PDI expression detected by immunofluorescence **(C)** and western blot **(D)** showed no difference between Wistar and SHR. Images are representative of three different experiments. Images were acquired using a 20× objective lens with exposure intensity adjusted relatively to 6 week-old Wistar. PDI expression was normalized to β-actin. Results are expressed as percentage of 6 week-old Wistar and represent mean ± SEM of *n* = 6 experiments. ^*^p < 0.05 vs. Wistar; ^+^*p* < 0.05 vs. Wistar 6 weeks.

Interestingly, PDI expression followed the same pattern of increase as Nox1, increasing in mesenteric resistance arteries from 8 to 12 week-old SHR (Figures 4A,B), while in aorta there was no difference among groups (Figures 4C,D). These data suggest a positive correlation between Nox-1/ PDI expression and ROS generation during the development of hypertension only in mesenteric resistance arteries.

**Figure 5:**
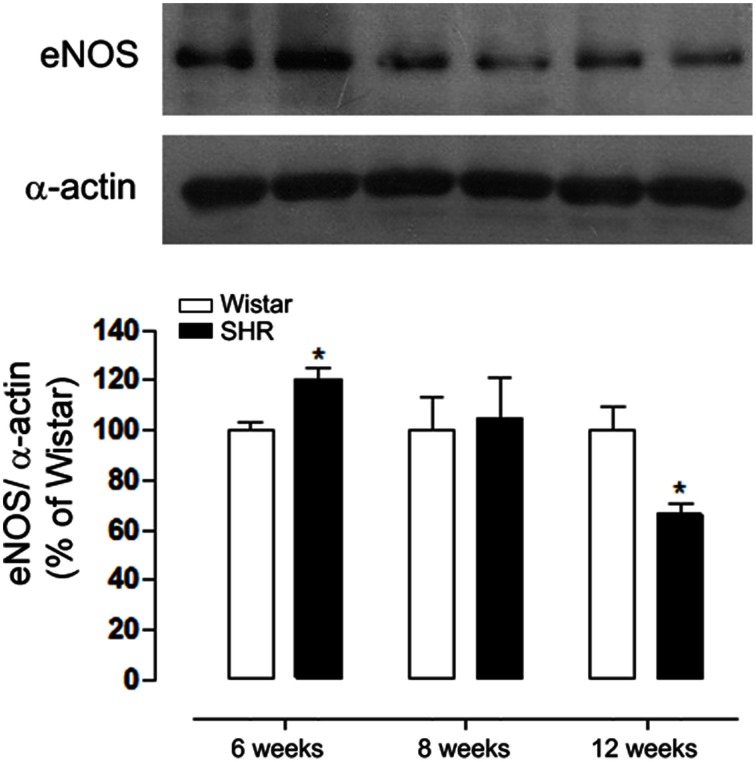
**Age-dependent differences in eNOS expression during the development of hypertension**. eNOS expression in aortas detected by western blot (WB) was increased in 6, did not change in 8 and decreased in 12 week-old SHR and compared to Wistar rats. Protein expression was normalized to α-actin. Upper panel shows a representative blot of five experiments. Results are expressed as percentage of respective Wistar and represent mean ± SEM. ^*^p < 0.05 vs. age-matched Wistar.

In order to verify protein expression of endothelial NO synthase (eNOS) during the development of hypertension we performed Western Blot experiments. Figure 5 shows eNOS protein expression is increased in 6 week, did not change in 8 week and decreased in 12 week SHR aortas as compared to Wistar rats.

### AT1 receptor antagonism reduces oxidative stress and Nox-1/ PDI expression in mesenteric resistance arteries

**Figure 6:**
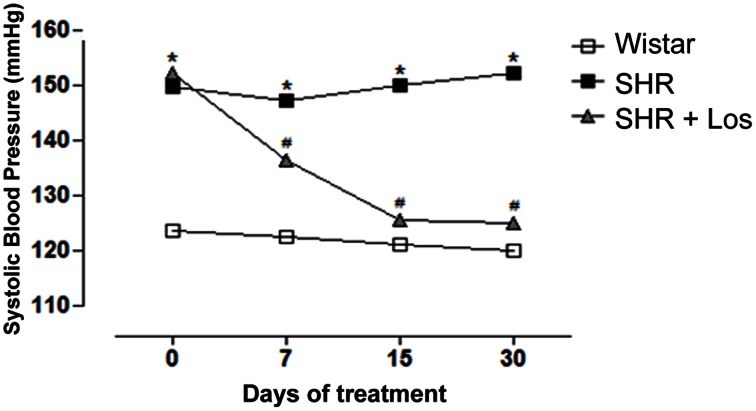
**AT1R antagonist decreases systolic blood pressure (SBP) in hypertensive rats**. Values of SBP measured by tail-cuff method in SHR and Wistar rats. A subgroup of 7 week-old SHR was treated with Losartan (Los, 20 mg/kg/day) during 30 days. Results are expressed as mean ± SEM of *n* = 5 rats. ^*^p < 0.05 vs. Wistar and ^#^*p* < 0.05 vs. SHR.

Losartan treatment was able to progressively decrease blood pressure in SHR to control levels (Figure 6).

**Figure 7:**
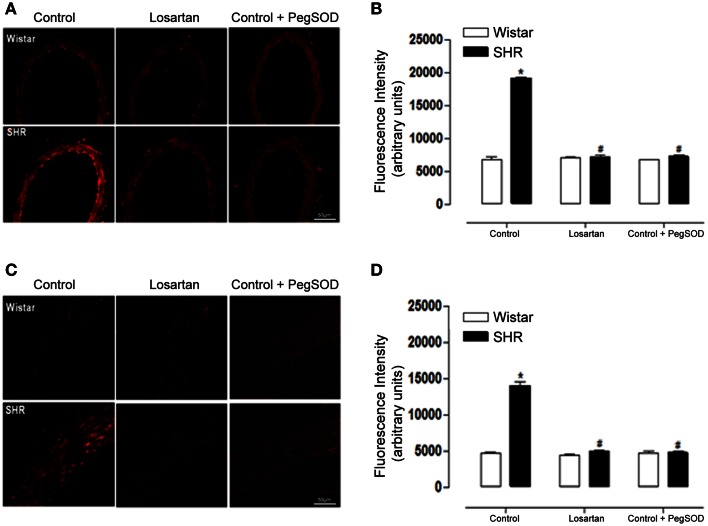
**AT1R antagonist decreases ROS generation in mesenteric resistance arteries and aorta from hypertensive rats**. Analysis of dihydroethidium (DHE) derived fluorescence **(A,C)** and quantification **(B,D)** show that losartan (20 mg/kg/day, 30 days) treatment reduces the enhanced ROS generation in mesenteric resistance arteries **(B)** and aorta **(D)** from SHR. A set of serial sections were treated with PEG SOD (150 U/mL) for 30 min before incubation with DHE and used as experiment control. Images are representative of three different experiments and were acquired using a 20× objective lens with exposure intensity adjusted relatively to Wistar non treated rats (control). Results are expressed as mean ± SEM of *n* = 3 experiments. ^*^p < 0.05 vs. Wistar; ^#^*p* < 0.05 vs. control SHR.

**Figure 8:**
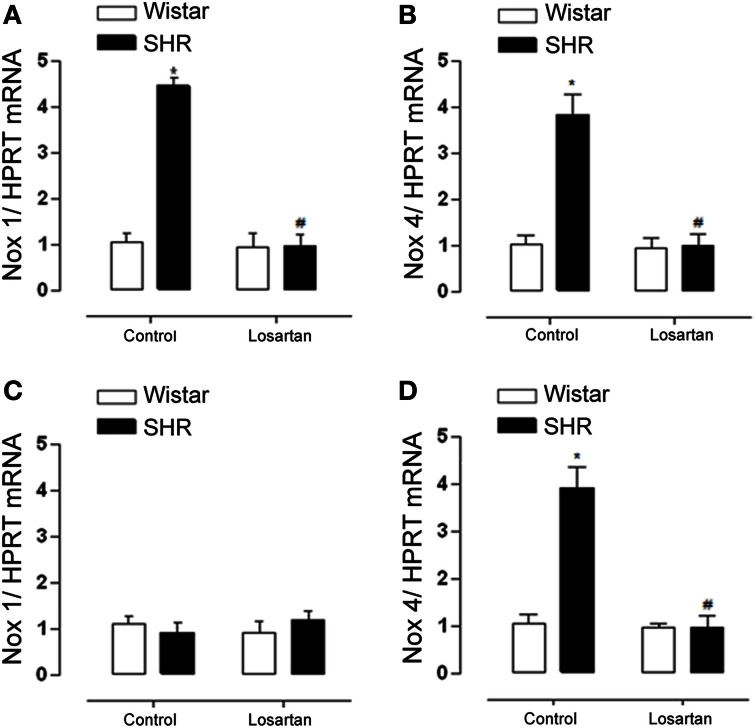
**AT1R antagonist decreases Nox1 expression only in resistance arteries and Nox 4 in both vessels from hypertensive rats**. Nox1 and Nox4 mRNA levels detected by RT-PCR. Nox1 mRNA expression was decreased in mesenteric resistance arteries **(A)** from SHR treated with losartan (20 mg/kg/day, 30 days), while in aorta **(C)** there was no change. Nox4 mRNA expression increased in both mesenteric arteries **(B)** and aorta **(D)** from SHR treated and this effect was reversed after Losartan treatment. Results were normalized to HPRT mRNA and are expressed as fold increase as compared to Wistar non treated rats (control). Data represent mean ± SEM of *n* = 5 experiments. ^*^p < 0.05 vs. Wistar, ^#^*p* < 0.05 vs. control SHR.

Furthermore, AT1 receptor antagonism blocked the increased ROS generation in both mesenteric resistance arteries (Figures 7A,B) and aorta (Figures 7C,D) of SHR. The incubation of the arterial segments with Peg-SOD decreased DHE fluorescence, which implicates superoxide as one of the oxidants generated in these vessels (Figures 7A–D). In addition, increased Nox1 gene expression observed only in SHR mesenteric resistance arteries was reversed by losartan treatment (Figures 8A,C). Nox4 gene expression increased in non treated (control) SHR mesenteric resistance arteries and aortas and AT1 antagonism blocked this effect in both vessels (Figures 8B,D, respectively).

**Figure 9:**
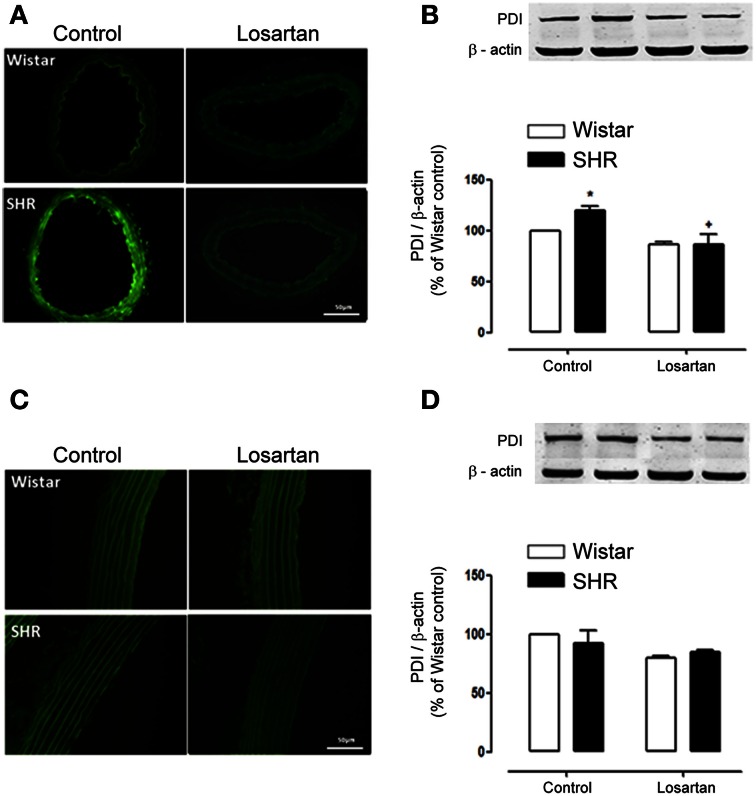
**Losartan treatment decreases PDI expression only in resistance arteries from hypertensive rats**. In mesenteric arteries losartan treatment (20 mg/kg/day, 30 days) decreased PDI expression in SHR as shown by immunofluorescence **(A)** and western blot **(B)**. In aorta, immunofluorescence **(C)** and western blot **(D)** showed no difference in PDI expression in Wistar and SHR before and after treatment with losartan. Images are representative of three different experiments and were acquired using a 20× objective lens with exposure intensity adjusted relatively to Wistar non treated rats (control). PDI expression was normalized to β-actin expression. Results are expressed as percentage of Wistar control and represent mean ± SEM of *n* = 4 experiments. ^*^p < 0.05 vs. control Wistar and ^+^*p* < 0.05 vs. control SHR.

Similarly to Nox1, the increased expression of PDI in mesenteric resistance arteries was reduced by losartan treatment (Figures 9A,B). However, neither hypertension nor losartan treatment changed PDI expression in aorta (Figures 9C,D). These results indicate a relationship between Nox1 and PDI in Ang II-induced ROS generation in SHR mesenteric resistance arteries.

### PDI inhibition decreases Ang II-induced redox signaling in resistance arteries

**Figure 10:**
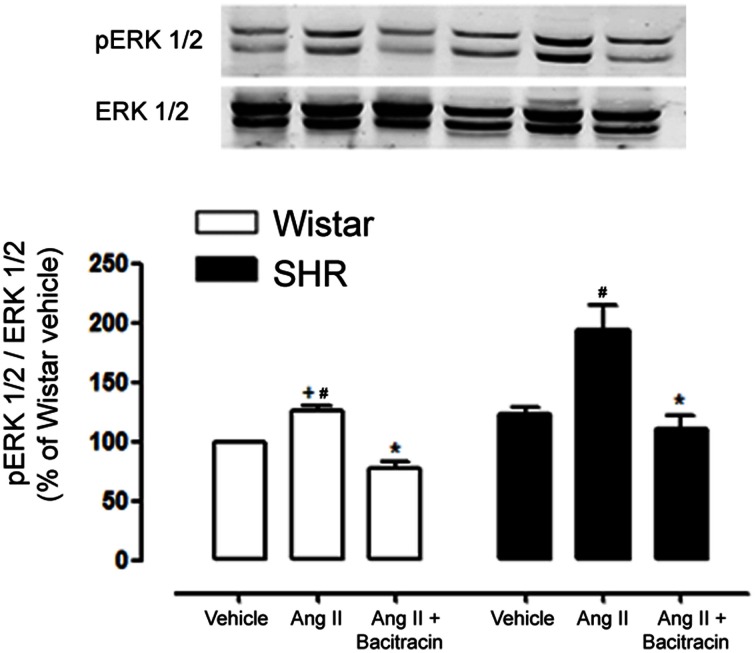
**PDI inhibition decreases Ang II induced ERK 1/2 activation in mesenteric resistance arteries of Wistar and SHR**. Ang II rapidly induced ERK 1/2 phosphorylation (p-ERK; 5 min) and this activation is increased in SHR mesenteric arteries as compared to Wistar. PDI inhibition (Bacitracin 0.5 mmol/L) significantly abolished p-ERK 1/2 in mesenteric arteries of both strains. Upper panel shows a representative blot for three experiments. Bar graph indicates relative quantification of p-ERK content expressed as the ratio between pERK and ERK 1/2 and normalized to the vehicle group, taken as 100%. Results are expressed as percentage of Wistar vehicle and represent mean ± SEM. ^*^p < 0.05 vs. respective Ang II; ^+^*p* < 0.05 vs. SHR Ang II; ^#^*p* < 0.05 vs. respective vehicle.

We then questioned whether PDI plays a role in Ang II redox signaling in mesenteric resistance arteries. Isolated mesenteric arteries from SHR and Wistar rats were acutely stimulated with Ang II (100 nmol/L) for 5 min in the presence of the PDI inhibitor bacitracin (0.5 mmol/L). An increased Ang II-induced phosphorylation of ERK 1/2 was observed in mesenteric resistance arteries of SHR when compared to Wistar rats. PDI inhibition significantly decreased ERK 1/2 activation by Ang II in both SHR and Wistar mesenteric arteries (Figure 10).

## Discussion

Previous studies from our group revealed a close relationship between PDI and NADPH oxidase. In particular in VSMCs, PDI associates with NADPH oxidase subunits (Janiszewski et al., 2005) regulates basal NADPH oxidase activity and Ang II-induced activity (Fernandes et al., 2009), and is involved in PDGF-induced Nox1-dependent migration (Pescatore et al., 2012). However, involvement of PDI in hypertension vascular dysfunction associated with increased NADPH oxidase activity has not been investigated. In this study we show for the first time that PDI expression increases during hypertension development in resistance but not in conductance arteries. Furthermore, we observed that the increase in PDI is accompanied by an increase in Nox1, but not Nox4, expression and that PDI plays a role in Ang II-induced redox signaling in hypertension.

Hypertension is widely known to be associated with high levels of ROS and an increase in Nox1 activity and expression. Nox1 transgenic mice show augmented ROS levels and increased pressor and hypertrophic responses to Ang-II. Therefore, Nox1-derived ROS could contribute to increased wall thickness in hypertension development (Dikalova et al., 2005). In the present study we used spontaneously hypertensive rat (SHR), a model of genetic hypertension. Our data confirms previous studies showing an increase in blood pressure (BP) from 6 to 12 weeks of age (Nabha et al., 2005). In addition, it has been shown that SHR exhibit high levels of ROS in the kidney only a few weeks after birth, before the rise in blood pressure or in inflammatory markers, which is suggestive that ROS enhancement precedes the increment in blood pressure (Chabrashvili et al., 2002). However, in the vasculature there is still some controversy. We investigated ROS generation, PDI and Nox expression in resistance (mesenteric arteries) and conductance arteries (aorta) in normotensive (6 week-old), pre-hypertensive (8 week-old) and hypertensive (12 week-old) SHR. We show here that ROS generation increases in mesenteric arteries concomitantly with the increase in blood pressure or else, after 8 weeks. In contrast, in aorta increased ROS generation is already present in 6 week-old SHR, despite normal blood pressure. Therefore our results confirm previous studies (Nabha et al., 2005) which show that at least in the aorta increase in ROS generation precedes the increase in blood pressure. Furthermore, we show that Nox isoform expression differed in resistance and conductance arteries during the establishment of hypertension. Nox1 was the source of ROS in resistance arteries and Nox4 in conductance ones. Additionally, our data demonstrate that only in mesenteric resistance arteries ROS generation seems to be Nox1 dependent. In fact, high expression of Nox1, and Nox4 was reported in VSMC isolated from mesenteric arteries of 12 week-old SHR but in accordance to our findings, only siNox1 inhibited ROS production in these cells (Briones and Touyz, 2010). In aorta, some authors show high levels of Nox1, 2, and 4 (Akasaki et al., 2006; Li et al., 2010), while others show higher expression of Nox1 and 2, but not Nox4 (Wind et al., 2010). Nevertheless, the age of these rats was either not informed or older than the ones used here.

Interestingly, PDI expression only increased in mesenteric arteries, and concomitantly to Nox1 expression, despite previous data from our group which showed that PDI associates with both Nox1 and Nox4 (Janiszewski et al., 2005). In fact, evidence of a closer relationship between Nox1 and PDI in VSMCs, was demonstrated recently. Overexpression of PDI in aortic VSMCs resulted in an increase in Nox1 expression, while silencing of PDI decreased Nox1 expression. In contrast, neither PDI overexpression nor silencing altered Nox4 expression in these cells (Fernandes et al., 2009). Furthermore, PDI also regulates Ang II and PDGF induced effects on Nox1 expression, while no effect was observed in Nox 4 (Fernandes et al., 2009; Pescatore et al., 2012). The present data corroborates these studies and provides a potential role for PDI in Nox1 derived ROS generation in hypertension. Therefore, although, the exact mechanisms regarding PDI-NADPH oxidase interaction remains to be elucidated, the present study is the first to provide evidence for the role of PDI in Nox1 signaling in hypertension.

Our data also show differential eNOS expression in aorta during development of hypertension. Particularly, we show an increase of eNOS in aorta in the early stages of the disease. Corroborating these data previous studies have shown that 3 week old SHR present an enhancement of aorta and kidney protein expression of eNOS and NO generation, suggesting that NO-pathway is upregulated in SHR before the onset of hypertension (Vaziri et al., 1998). On the other hand, the present data demonstrated a reduction of eNOS expression in aorta of 12 week old SHR. In this context, the literature shows contradictory results of eNOS expression in aorta of SHR after established hypertension. Indeed this expression has been shown to be enhanced (Vaziri et al., 1998) or reduced (Rodriguez-Rodriguez et al., 2007; Xu and Liu, 2013). Also, eNOS activation is decreased (Filho et al., 2013) and ROS production in aorta of SHR was associated with eNOS uncoupling (Li et al., 2006). Taken together, the increase in Nox1 and Nox4 and the reduction of eNOS expression in 12 week old arteries from SHR could be associated with the maintenance of hypertension.

Ang II signaling through Nox derived ROS can activate MAP kinases, tyrosine kinases, transcription factors and modulate intracellular free Ca^2+^ concentration ([Ca^2+^]i), participating in processes like growth, migration, deposition of extracellular matrix and inflammation (Knock and Ward, 2011). In order to investigate the effect of Ang II on PDI expression, pre hypertensive, 7 week-old SHR were treated with AT1 receptor antagonist. Treatment with losartan decreased blood pressure and ROS generation in both mesenteric resistance arteries and aorta. However, only in SHR mesenteric arteries losartan treatment lowered PDI and Nox1 expression to control levels. It is known that antagonism of AT1 receptor can lower ROS generation and expression of several NADPH oxidase subunits in SHR (Zalba et al., 2000; Akasaki et al., 2006), however this is the first report that Ang II increases PDI expression during the development of hypertension.

To further investigate the mechanisms involved in this effect, mesenteric resistance arteries from Wistar and SHR were isolated and stimulated with Ang II in the presence of PDI non specific inhibitor bacitracin. PDI inhibition resulted in a reduction in Ang II induced ERK 1/2 phosphorylation in both strains. It has been shown that in VSMCs from mesenteric arteries of SHR, the increase in ROS generation is involved in the increase of EGFR transactivation and as a consequence, in increased ERK 1/2 phosphorylation induced by Ang II (Li et al., 2010). Furthermore, we have shown that PDI silencing decreases Ang II redox signaling in aortic VSMCs (Janiszewski et al., 2005) and TNF-α signaling in endothelial cells (Camargo Lde et al., 2013). Altogether our results suggest a role for PDI in Ang II increased signaling in resistance vessels from SHR rats.

One interesting finding of this study is that SHR mesenteric arteries presented higher expression of PDI. It has been reported that increase in one subunit is accompanied by increase in other subunits of NADPH oxidase (Fukui et al., 1997; Dikalova et al., 2005). We have previously shown that PDI colocalizes with NADPH subunits (Janiszewski et al., 2005) and the increased expression of this protein in SHR reinforces the role of PDI as a regulatory protein of the oxidase complex. Moreover, Fernandes et al. (2009) demonstrated that overexpression of PDI in rabbit aortic smooth muscle cells resulted in preemptive activation of NADPH oxidase with enhanced production of ROS. Therefore, in the present study increased PDI expression in resistance mesenteric arteries could account for increased Nox1 expression and activity, and consequently higher ROS generation and Ang II responses in these vessels.

Altogether our results show that PDI expression increases concomitantly to Nox1 in resistance arteries during hypertension development and that this effect is dependent on Ang II signaling through the AT1 receptor. Therefore, PDI could be a novel regulator of Nox1 signaling in resistance arteries contributing to oxidative stress and vascular dysfunction in hypertension.

### Conflict of interest statement

The authors declare that the research was conducted in the absence of any commercial or financial relationships that could be construed as a potential conflict of interest.
